# Total hip arthroplasty versus hemiarthroplasty for displaced femoral neck fractures in the healthy elderly: a meta-analysis and systematic review of randomized trials

**DOI:** 10.1007/s00264-012-1569-7

**Published:** 2012-05-24

**Authors:** Paul T. P. W. Burgers, Arnoud R. Van Geene, Michel P. J. Van den Bekerom, Esther M. M. Van Lieshout, Bastiaan Blom, Ilyas S. Aleem, M. Bhandari, Rudolf W. Poolman

**Affiliations:** 1 Department of Surgery-Traumatology, Erasmus MC, Rotterdam, The Netherlands; 2 Joint Research, Department of Orthopaedic Surgery, Onze Lieve Vrouwe Gasthuis, Amsterdam, The Netherlands; 3 Department of Orthopaedic Surgery, Spaarne Ziekenhuis, Hoofddorp, The Netherlands; 4 Division of Orthopaedic Surgery, Department of Surgery, University of Toronto, Torronto, ON Canada; 5 Division of Orthopaedic Surgery, Department of Surgery, McMaster University, Hamilton, ON Canada

## Abstract

**Purpose:**

Displaced femoral neck fractures in healthy elderly patients have traditionally been managed with hemiarthroplasty (HA). Recent data suggest that total hip arthroplasty (THA) may be a better alternative.

**Methods:**

A systematic review of the English literature was conducted. Randomized controlled trials comparing all forms of THA with HA were included. Three authors independently extracted articles and predefined data. Results were pooled using a random effects model.

**Results:**

Eight trials totalling 986 patients were retrieved. After THA 4 % underwent revision surgery versus 7 % after HA. The one-year mortality was equal in both groups: 13 % (THA) versus 15 % (HA). Dislocation rates were 9 % after THA versus 3 % after HA. Equal rates were found for major (25 % in THA versus 24 % in HA) and minor complications (13 % THA versus 14 % HA). The weighted mean of the Harris hip score was 81 points after THA versus 77 after HA. The subdomain pain of the HHS (weighted mean score after THA was 42 versus 39 points for HA), the rate of patients reporting mild to no pain (75 % after THA versus 56 % after HA) and the score of WOMAC (94 points for THA versus 78 for HA) all favored THA. Quality of life measured with the EQ-5D favored THA (0.69 versus 0.57).

**Conclusions:**

Total hip arthroplasty for displaced femoral neck fractures in the fit elderly may lead to higher patient-based outcomes but has higher dislocation rates compared with hemiarthroplasty. Further high-quality randomized clinical trails are needed to provide robust evidence and to definitively answer this clinical question.

## Introduction

The optimal surgical management of displaced femoral neck fractures in the elderly is the subject of an ongoing scientific and clinical debate [1, 2]. About 50 % of the total hip fracture population has a displaced femoral neck fracture. Determining the optimal therapy is important as in the year 2000 an estimated 1.6 million hip fractures occurred [3], and this incidence is expected to increase to over six million hip fractures worldwide by the year 2050 [4]. Reported causes are the changing demography and an increasing contribution of developing countries [5].

Patients with a hip fracture have high mortality and disability [6]. As a consequence these fractures have a significant impact both on the patients’ personal dependence, mobility, and quality of life as well as on global economic health costs. Especially, the one-year mortality after a femoral neck fracture, even in selected patients, ranges from 14 % to 36 % [7], so the actual numbers are even higher. Moreover, worldwide 4.5 million persons are living with disability from hip fractures yearly. This number is expected to increase to 21 million persons in the next 40 years. The costs of treating a hip-fracture patient are about three times higher than those of caring for a patient without a fracture [8]. The worldwide direct and indirect annual costs of hip fractures in 1990 were estimated at US$34.8 billion [9].

Hemiarthroplasty (HA) and total hip arthroplasty (THA) remain as widely accepted methods of hip replacement after fracture. In the long run some patients treated with HA require conversion to THA because of activity limiting thigh pain due to acetabulum wear. Reported advantages of HA compared with THA are reduced dislocation rates, less complex surgery, shorter operation times, less blood loss, and lower initial costs [10]. Therefore, a number of authors prefer HA for displaced femoral neck fractures [11–13]. In contrast, evidence is accumulating to support better function and superior patient satisfaction for patients treated with THA [10, 14–17]. Consequently, after weighing the pros and cons other authors advocate THA as preferable treatment for displaced fractures in the elderly [18–20].

In two previous systematic reviews [2, 21] it was concluded that large well-designed randomized trials are needed in order to draw a definitive conclusion as the scientific evidence is still insufficient. Since the publication of these reviews, data of the largest trial (*N* = 250) [13] became available; these are included in the present study.

The aim of the present study was to conduct a systematic review and meta-analysis using the best available evidence in order to determine primarily the outcomes of reoperations; secondary outcomes were dislocation rates, mortality rates, complications, function, and pain of total hip arthroplasty versus hemiarthroplasty for displaced femoral neck fractures in the healthy elderly.

## Materials and methods

The present review and meta-analysis were reported according to the PRISMA statement [22]. Methods used for the analysis, search strategy, and inclusion criteria were specified in advance and documented in an unpublished protocol.

### Search strategy

An electronic search of the literature was independently performed in duplicate by two clinical librarians at different time points from inception to February 22, 2011 in the following databases: MEDLINE (PubMed), EMBASE, World of Science and Cochrane Central Register of Controlled Trials. The electronic search was individually tailored to each database aiming at maximizing the sensitivity of the search when identifying studies having terms relevant to “hemiarthroplasty”, “total hip arthroplasty” and “intracapsular hip fracture.” The complete search terms are shown in Appendix 1. In addition, bibliographies were reviewed of all selected full text articles to identify additional articles. In order to evaluate any ongoing randomized trials, the international trial registries (www.clinicaltrials.gov, www.trialregister.nl and www.apps.who.int/trialsearch) were accessed (last visit: March 11, 2011).

### Eligibility criteria

Three reviewers (PTPWB, ARG and BB) independently identified titles and abstracts relevant to total hip arthroplasty versus hemiarthroplasty for dislocated femoral neck fractures. Full text published articles and unpublished data of completely finished and analysed studies were included. Authors of studies for which only the abstract was available were contacted for availability of study data. The following eligibility criteria had to be met: (1) use of (quasi) random allocation of treatments, (2) patients aged 50 years or older with a displaced femoral neck fracture, (3) inclusion of a treatment arm receiving any form of hemiarthroplasty, (4) inclusion of a treatment arm receiving any form of total hip arthroplasty, and finally all papers had to report data on the primary outcome, being revision surgery. No restrictions related to the length of follow-up or languages were defined. The reviewers obtained consensus on inclusion status with any found discrepancies.

The primary endpoint was defined as revision surgery within the different study periods. Secondary outcomes were mortality, dislocation, major and minor complications, functional outcome, pain, and quality of life. The minor and major complications were arbitrarily defined by two authors (PTPWB and ARG) as specified in Appendix 2.

### Data extraction and analysis

Three reviewers (PTPWB, ARG and BB) independently extracted the inclusion criteria data from each study meeting. Data included demographics, methodology, details on intervention, and reported outcomes. Data for the primary and secondary outcomes were extracted and collected on a predefined standardized electronic data collection form. In case of differences, the reviewers discussed this item in order to meet consensus; if no agreement could be reached, a third author (RWP) decided. Methodological study quality was gauged by noting the specifics of randomization, concealment of allocation, blinding, adherence to the intention to treat principle and the extent of follow-up (Table 2) [23].

Review Manager software (RevMan Version 5.0.22, Copenhagen: The Nordic Cochrane Centre, The Cochrane Collaboration, 2008.) was used for statistical analysis and for generating figures. For combining the results found in the different trials the statistical method of Mantel-Haenszel with random effects method was used for dichotomous outcomes, and risk ratios for THA compared with HA were calculated. For continuous outcomes the statistical inverse variance method was used with random effects analysis model and mean differences were calculated. Heterogeneity between studies was assessed by using I2 statistics. The quality of the individual parameters was assessed with Grade profiler software (GRADEpro. Version 3.2.2. for Windows. Jan Brozek, Andrew Oxman, Holger Schünemann, 2008) [24].

## Results

**Fig. 1:**
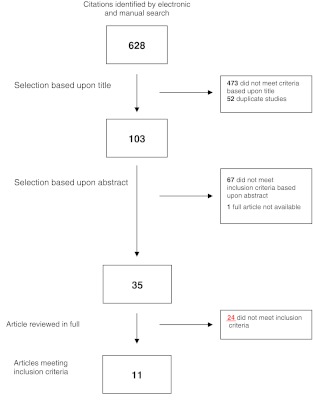
Flow chart of article selection process

After applying the search strings 628 potentially eligible articles were identified, of which 473 were excluded based upon title, and 52 studies were duplicates of these reports. Another 67 manuscripts were excluded after reviewing the abstract. Contact with the author of one abstract revealed that the trial was still actively recruiting patients. In the next phase of the selection procedure 35 full articles were reviewed of which 24 articles did not meet the predefined eligibility criteria. Two studies were published twice [10, 16, 25, 26]. One report was considered the index report, the other article was searched for additional information. Data from both articles were included in this study. One manuscript was a 13-year follow-up [27] of a previously conducted RCT [28]. Data from both reports were included in the analysis. In conclusion, a total of 11 articles about eight studies were included for the present review and meta-analysis which involved a total of 986 patients [13–17, 25–29] (Fig. 1).

Tables 1, 2, 3 and 4 summarize the methodological quality, the methodological characteristics, the characteristics of the interventions and the characteristics of individual studies. Two studies had also included a third (internal fixation) arm [17, 27, 28]. These data were not taken into account, as internal fixation was not assessed in the present study. In all studies inclusion and exclusion criteria were clearly defined prior to the study in order to select patients with an ambulatory and cognitive fit pre-fracture status. The quality of the individual parameters ranged from low to very low (Table 1). In three studies, sealed envelopes were used as randomization system [14–16, 26]; one of which was stated as block randomization [16]. A fully automated computerized allocation system was used in two studies [10, 13]. Other methods used for treatment allocation were by hospital number [29], fixed treatment sequence [28], and according to the order of admission [17]. The outcome assessor was blinded for the allocated treatment in only one study [17]. Patients were not blinded for treatment in any of the studies. Three studies [10, 15, 16, 25, 26] stated an intention to treat analysis, one a per protocol analysis [13] and four studies did not specify the data analysis method [14, 17, 27–29]. For all eight studies [13–17, 25–29] the follow-up period was at least one year (Table 2). All patients in the THA arm were treated with a cemented stem, except in one study [16] where both cemented and uncemented stems were used. For patients treated with hemiarthroplasty in two studies [16, 29] both cemented and uncemented stems were used; in one study [17] cementing of the stem was not specified. In four studies cemented stems were used; in one study uncemented stems were used. In three studies [13, 14, 28] only unipolar heads were used, in three studies [10, 15, 29] only bipolar heads were used, in one study [16] both types of heads were used and one study [17] did not specify the polarity of the head component of the hemiarthroplasty (Table 3).

**Table 1:** Quality assessment and summary of findings

Quality assessment							Summary of findings					Importance
							No. of patients		Effect		Quality	
No. of studies	Design	Limitations	Inconsistency	Indirectness	Imprecision	Other considerations	Total hip	Hemiarthroplasty	Relative risk (95 % CI)	Absolute		
One-year mortality												
6 ^a^	Randomized trials	Very serious^b^	No serious inconsistency ^c^	Serious^d^	Serious^a,e^	None^c^	53/393 (13.5 %)	64/423 (15.1 %)	RR 0.91 (0.65–1.27)	14 fewer per 1000 (from 53 fewer to 41 more)	Very low	Critical
								13.6 %		12 fewer per 1000 (from 48 fewer to 37 more)		
Revision surgery												
8 ^c^	Randomized trials	Very serious^b^	No serious inconsistency ^c^	Serious^d^	Serious^f^	None^c^	19/472 (4 %)	36/514 (7 %)	RR 0.59 (0.32–1.09)	29 fewer per 1000 (from 48 fewer to 6 more)	Very low	Important
								7.1 %		29 fewer per 1000 (from 48 fewer to 6 more)		
Dislocation												
6 ^g^	Randomized trials	Very serious^b^	No serious inconsistency ^c^	Serious^d^	Serious^h,i^	None^c^	33/369 (8.9 %)	14/411 (3.4 %)	RR 2.53 (1.05–6.1)	52 more per 1000 (from 2 more to 174 more)	Very low	Important
								0 %		0 more per 1000 (from 0 more to 0 more)		
Major complications												
5 ^j^	Randomized trials	Very serious^b^	No serious inconsistency ^c^	Serious^d^	Serious^j,k^	None^c^	76/302 (25.2 %)	80/330 (24.2 %)	RR 1.07 (0.76–1.5)	17 more per 1000 (from 58 fewer to 121 more)	Very low	Important
								8.2 %		6 more per 1000 (from 20 fewer to 41 more)		
Minor complication												
5 ^l^	Randomized trials	Very serious^b^	No serious inconsistency^3^	Serious^d^	Serious^l,m^	None^c^	38/302 (12.6 %)	45/330 (13.6 %)	See comment	10 fewer per 1000 (from 60 fewer to 40 more)	Very low	Important
								7 %		5 fewer per 1000 (from 31 fewer to 20 more)		

*CI* confidence interval, *RR* relative risk

^a^ Two out of eight studies did not adequately provide number of deaths after one-year follow-up

^b^ Allocation concealment: 3/8 study used sealed envelopes, 1/8 hospital number, 2/8 computerized, 1/8 order of admission, 1/8 did not specify blinding: none of studies blinded the patients, only 3/8 studies report on a blinded outcome assessor failure to adhere to the intention to treat principle: 5/8 studies

^c^ No explanation was provided

^d^ In the different trials, different approaches and materials, e.g. cement vs uncemented were used. This may have had some effect, e.g. pain, function or dislocation

^e^ Total (cumulative) sample (size =117) is lower than the calculated optimal information size (OIS) (64/423 = 0.15-- > needed: RR 25 %: 500)

^f^ Total (cumulative) sample (size =55) is lower than the calculated optimal information size (OIS) (36/514 = 0.07-- > needed: RR 5%: 600)

^g^ Two out of eight studies did not adequately provide information on dislocation rates

^h^ Two out of eight studies did not provide clear numbers of dislocation at all

^i^ Total (cumulative) sample (size =47) is lower than the calculated optimal information size (OIS) (14/411 = 0.03-- > needed: RR 25%: 600)

^j^ Three out of eight studies did not adequately provide information on major complications

^k^ Total (cumulative) sample (size = 156) is lower than the calculated optimal information size (OIS) (45/330 = 0.24-- > needed: RR 5%: 500)

^l^ Three out of eight studies did not adequately provide information on minor complications

^m^ Total (cumulative) sample (size =83) is lower than the calculated optimal information size (OIS) (45/330 = 0.1-- > needed: RR 5 %: 500)

**Table 2:** Methodological characteristics of individual selected studies

Study	Type of randomization	Allocation concealment	Patient blinding	Intention to treat	Follow-up period (years)
Baker et al. [14]	Sealed envelopes	NS	No	NS	3
Blomfeldt et a. [15]	Sealed envelopes	No	No	Yes	1
Dorr et al. [29]	Hospital number	No	No	NS	4
Keating et al. [25]	Computerized	No	No	Yes	2
Macaulay et al. [16, 26]	Sealed envelopes	NS	No	Yes	2
Mouzopoulos et al. [17]	Order of admission	Yes	No	NS	4
Skinner et al. [28]	Day of the week	No	No	NS	1
Van den Bekerom et al. [13]	Computerized	No	No	Per protocol	5

*NS* not specified

**Table 3:** Intervention characteristics of individual selected studies

Study	THA	HA	Type	Surgical approach	Surgeon’s grade
Baker et al. [14]	Cemented	Cemented	Unipolar	Lateral	Staff and residents
Blomfeldt et a. [15]	Cemented	Cemented	Bipolar	Anterolateral^a^	Staff
Dorr et al. [29]	Cemented	Cemented or uncemented	Bipolar	Posterior	NS
Keating et al. [25]	Cemented	Cemented	Bipolar	Posterior or lateral	Staff, residents and SHO
Macaulay et al. [16, 26]	Cemented or uncemented	Cemented or uncemented	Uni- or bipolar	Posterolateral or anterolateral^a^	Staff and fellows
Mouzopoulos et al. [17]	Cemented	NS	NS	NS	NS
Skinner et al. [28]	Cemented	Uncemented	Unipolar	Posterolateral	Registrars and consultants and SHO’s
Van den Bekerom et al. [13]	Cemented	Cemented	Unipolar	Posterolateral, (antero)lateral	Staff and residents

*THA* total hip arthroplasty, *HA* hemi arthroplasty, *NS* Not specified, *SHO* senior house officers

^a^ via Modified Hardinge

**Table 4:** Study characteristics of individual selected studies

Study	Recruitment period	THA number (*N*)	HA number (*N*)	Single-/ multicenter (*N* sites)	THA mean age	HA mean age
Baker et al. [14]	NS	40	41	Multi-center (3)	74	76
Blomfeldt et a. [15]	NS	60	60	Single center	81	81
Dorr et al. [29]	March 1980 to July 1982	39	50	Single center	69	
Keating et al. [25]	Sep 1996 to June 2000	69	69	Multi-center (11)	75	75
Macaulay et al. [16, 26]	18 months (NS)	17	23	Multi-center (5)	82	77
Mouzopoulos et al. [17]	April 1999 to April 2002	43	43	Multi-center (NS)	73	74
Skinner et al. [28]	Dec 1984 to Dec 1986	89	91	Single center	81	82
Van den Bekerom et al. [13]	Jan 1995 to Dec 2001	115	137	Multi-center (8)	82	80

*THA* total hip arthroplasty, *HA* hemi arthroplasty, *NS* not specified

The exact recruitment period was not specified in three studies [14–16]. The number of patients per arm ranged from 17 to 137. Three studies [15, 28, 29] used a single-center design; five studies [10, 13, 14, 16, 17] were performed with a multicenter approach (Table 4).

### Clinical outcomes

#### Revision surgery

**Fig. 2:**
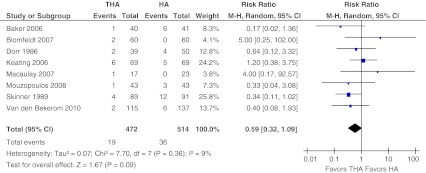
Revision surgery. Forest plot comparing risk ratios of revision and planned revision surgery after total hip arthroplasty versus hemiarthroplasty in displaced femoral neck fractures in the healthy elderly. Mantel-Haenszel statistical method was used with the ‘random effects’ analysis method for dichotomous data. *M-H* Mantel-Haenszel, *THA* total hip arthroplasty, *HA* hemiarthroplasty

Data on revision surgery and reported planned revision surgery were pooled, totaling 986 patients and 55 events (5 %). Revision surgery was performed in 4 % in the THA-arm versus 7 % in the HA-arm (Fig. 2). There was low evidence of heterogeneity across the studies (I2 = 9 %, *P* = 0.36). No statistically significant difference in revision surgery between the two groups (relative risk, RR 0.59, 95 % confidence interval CI 0.32–1.09, absolute risk difference, ARD −0.02, 95 % CI −0.06 to 0.01) could be found. However, the pooled data showed a trend towards less revision surgery for patients who had undergone total hip arthroplasty compared with those who had undergone hemiarthroplasty.

#### One-year mortality

**Fig. 3:**
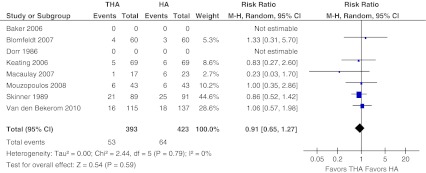
One year mortality. Forest plot comparing risk ratios of one-year mortality after total hip arthroplasty versus hemiarthroplasty in displaced femoral neck fractures in the healthy elderly. Mantel-Haenszel statistical method was used with the ‘random effects’ analysis method for dichotomous data. *M-H* Mantel-Haenszel, *THA* total hip arthroplasty, *HA* hemiarthroplasty

Data for mortality at one year were pooled. Six out of the eight selected studies provided adequate data on one-year mortality [10, 13, 15–17, 28] which involved a total of 816 patients and 117 deaths (overall 14 %; Fig. 3). The one-year mortality was 13 % in the THA-arm versus 15 % in the HA-arm. There was no evidence of heterogeneity (I2 = 0 %, *P* = 0.79). The pooled one-year mortality data did not differ between patients who had undergone total hip arthroplasty or hemiarthroplasty (RR 0.91, 95 % CI, 0.65–1.27, ARD −0.01, 95 % CI −0.05 to 0.03).

#### Dislocation

**Fig. 4:**
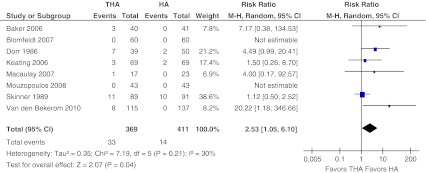
Dislocation. Forest plot comparing risk ratios of dislocation after total hip arthroplasty versus hemiarthroplasty in displaced femoral neck fractures in the healthy elderly. Mantel-Haenszel statistical method was used with the ‘random effects’ analysis method for dichotomous data. *M-H* Mantel-Haenszel, *THA* total hip arthroplasty, *HA* hemiarthroplasty

Six of the included studies provided data on dislocation [10, 13, 14, 16, 28, 29] (Fig. 4). Another study did not report on dislocation [17], and one study reported that in both treatment arms there were no cases of dislocation [15]. The risk of dislocation was 9 % in the THA-arm versus 3 % in the HA-arm. There was low evidence of heterogeneity across the studies (I2 = 30 %, *P* = 0.21). Pooling the data of these 780 patients and 47 events (6 %) revealed a significant risk for dislocation after treatment with total hip arthroplasty for dislocated femoral neck fractures (RR 2.53, 95 % CI 1.05–6.10, ARD 0.05, 95 % CI 0.02–0.08).

### Complications (Appendix 2)

**Fig. 5:**
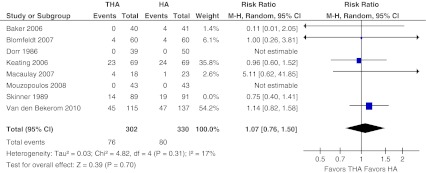
Major complications. Forest plot comparing risk ratios of minor complications (as defined in Appendix 2) after total hip arthroplasty versus hemiarthroplasty in displaced femoral neck fractures in the healthy elderly. Mantel-Haenszel statistical method was used with the ‘random effects’ analysis method for dichotomous data. *M-H* Mantel-Haenszel, *THA* total hip arthroplasty, *HA* hemiarthroplasty

Data on major complications were retrieved from five studies [10, 13–16] (Fig. 5). In addition, one study reported data on both minor and major complications, and these data had to be excluded as these were not specified to both treatment groups [29]. The outcome measures of two other studies were focused on functional recovery only and data on general complications were not presented [17, 28]. In 25 % major complications were found after THA versus 24 % after performing HA. No significant difference in major complication rates was found after either form of arthroplasty (RR 1.07, 95 % CI 0.76–1.50, ARD 0.00 95 % CI −0.08 to 0.08). Heterogeneity across the studies was 17 % (*P* = 0.31).

**Fig. 6:**
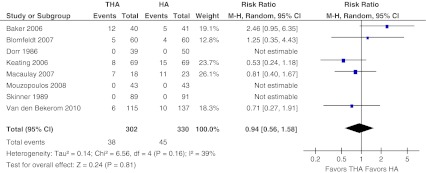
Minor complications. Forest plot comparing risk ratios of major complications (as defined in Appendix 2) after total hip arthroplasty versus hemiarthroplasty in displaced femoral neck fractures in the healthy elderly. Mantel-Haenszel statistical method was used with the ‘random effects’ analysis method for dichotomous data. *M-H* Mantel-Haenszel, *THA* total hip arthroplasty, *HA* hemiarthroplasty

The same five studies described in the section above on major complications presented data on general minor complications [10, 13–16] (Fig. 6). Heterogeneity across the five studies was 39 % (*P* = 0.16). In 13 % minor complications were found after THA versus 14 % after performing HA. After excluding the mentioned three studies for analysis, pooled data for general complications showed no significant difference in general minor complications (RR 0.94, 95 % CI 0.56–1.58, ARD −0.01, 95 % CI −0.08 to 0.07).

### Functional outcome

**Fig. 7:**
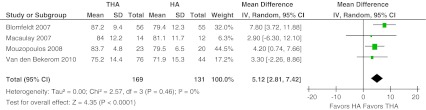
Harris hip score. Forest plot comparing risk ratios of total Harris hip score after total hip arthroplasty versus hemiarthroplasty in displaced femoral neck fractures in the healthy elderly. Inverse variance statistical method was used with the ‘random effects’ analysis method for continuous data. *IV* inverse variance,*THA* total hip arthroplasty, *HA* hemiarthroplasty

Four studies reported the Harris hip score after total follow-up [13, 15–17]. The Harris hip score ranges from 0 to 100 points and include function, pain, deformity and the range of motion. The weighted mean HHS was 81 (weighted mean SD 11) versus 77 (12) for THA and HA, respectively. A difference was found for the total score of this specific hip score (mean difference, MD 5.12, 95 % CI 2.81–7.42). Patients treated with THA reported statistically significantly higher Harris hip Scores. Heterogeneity across the studies was 0 % (*P* = 0.46) (Fig. 7).

### Pain

**Fig. 8:**
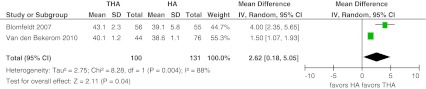
Harris hip score subdomain pain. Forest plot comparing risk ratios of Harris hip score pain section after total hip arthroplasty versus hemiarthroplasty in displaced femoral neck fractures in the healthy elderly. Inverse variance statistical method was used with the ‘random effects’ analysis method for continuous data. *IV* inverse variance,*THA* total hip arthroplasty, *HA* hemiarthroplasty

From two papers it was possible to calculate separately the pain subdomain of the Harris hip score [13, 15]. The weighted mean score for the pain subdomain of the HHS was 42 (weighted mean SD 2) versus 39 (3) for THA and HA, respectively. A significant difference was found favouring this score after treatment with THA (MD 2.62, 95 %CI 0.18–5.05) (Fig. 8).

**Fig. 9:**
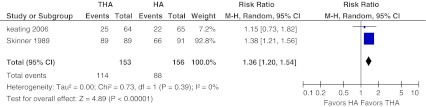
No to mild pain. Forest plot comparing risk ratios of no-to-mild pain after total hip arthroplasty versus hemiarthroplasty in displaced femoral neck fractures in the healthy elderly. Mantel-Haenszel statistical method was used with the ‘random effects’ analysis method for dichotomous data. *M-H* Mantel-Haenszel, *THA* total hip arthroplasty, *HA* hemiarthroplasty

Two studies [10, 28] reported pain in categories mild to no pain (with no analgesia) after total follow-up. No to mild pain was reported in 75 % after THA and in 56 % after HA. These pooled data also showed a significant difference in favour of the THA group (RR 1.36, 95 % CI 1.20–1.54. Heterogeneity across studies was 0 % (*P* = 0.39) (Fig. 9).

**Fig. 10:**
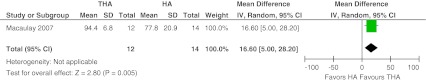
WOMAC subdomain pain. Forest plot comparing risk ratios of the Western Ontario and McMaster Universities Osteoarthritis Index questionnaire (WOMAC) pain score after total hip arthroplasty versus hemiarthroplasty in displaced femoral neck fractures in the healthy elderly. Inverse variance statistical method was used with the ‘random effects’ analysis method for continuous data. *IV* inverse variance,*THA* total hip arthroplasty, *HA* hemiarthroplasty

One study [16] separately showed the results of pain as scored with the Western Ontario and McMaster Universities Osteoarthritis Index questionnaire (WOMAC). The calculated mean difference was 16.60 points (THA 94.4, SD 6.8 versus HA 77.8, SD 20.9; 95 % CI 5.00–28.20, *P* = 0.005) favouring THA (Fig. 10).

### Quality of life

**Fig. 11:**
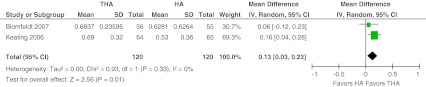
Quality of Life EQ5D. Forest plot comparing risk ratios of quality of life derived from the EuroQol-5 Dimensions (EQ-5D) after total hip arthroplasty versus hemiarthroplasty in displaced femoral neck fractures in the healthy elderly. Inverse variance statistical method was used with the ‘random effects’ analysis method for continuous data. *IV* inverse variance,*THA* total hip arthroplasty, *HA* hemiarthroplasty

Two European studies measured the quality of life with the EuroQol-5 Dimensions questionnaire at the final follow-up at one and two years respectively [10, 15]. The weighted mean EQ-5D score was 0.69 (weighted mean SD 0.28) versus 0.57 (0.48) for THA and HA, respectively. A difference was found favouring THA (MD 0.13, 95 % CI 0.03–0.23, *P* = 0.01). Heterogeneity across the studies was 0 % (*P* = 0.33) (Fig. 11).

## Discussion

Revision surgery rates and mortality rates were similar after THA and HA treatment for displaced femoral neck fractures in healthy elderly. None of these treatment options appeared to be superior with respect to postoperative minor or major complications. Risk of dislocation favoured HA. Estimates for function, pain and quality of life are less clear, but tend to be in favour of THA.

The first debate on the management of selected displaced hip fractures started in the 70s and the question is still valid, as is illustrated by the flow of publications with expert opinions, experiences and reviews. In the last three years two systematic reviews were published [21, 30], and the Cochrane review was recently updated [2], yet the question has still not been resolved.

Goh et al. performed a meta-analysis published in 2007 including three studies totaling 407 patients [10, 28, 29]. In summary, no differences were found for revision surgery, mortality and dislocation rates. Significantly less pain was reported for patients with THA after one year of follow-up. It was concluded that for a subgroup of healthy patients with a good prefracture mobility THA might be considered as primary surgical treatment [30].

Hopley et al. concluded in their extensive analysis with four randomized, three quasi-randomized and eight retrospective cohort studies that patients treated with total hip arthroplasty for intracapsular hip fractures may obtain better outcomes than those treated with HA [21]. In addition, they concluded that advantages with THA must be traded off against a slightly higher risk of dislocations and general complications.

From the latest Cochrane review on this topic including the same seven randomized trials as in the article by Hopley et al. it was concluded that although dislocation was more common with THA, there was a general trend towards better functional outcome scores for those treated with THA [2].

Data from the “ARTHRO trial” [13] were not included in the above-mentioned manuscripts. Beyond revision outcomes, this methodological well-designed trial provided new data on functional outcomes not previously available. Adding data from the 250 patients from this trial resulted in a 34 % increase in total population from randomized trials. The present analysis provides important new insights. First, our estimates of functional outcomes and pain suggest that patient-based results after THA may be better than that reported in previous meta-analyses. Also, our estimate of the difference in dislocation rates is less pronounced than previously reported. The overall mortality rate of 14 % as found in this study is lower than the frequently reported 20–25 %; this may be due to the relatively healthy patients that were included in the individual trials.

### Study limitations

The present review has some limitations. The published individual trials were generally of low methodological quality (I). For example, the methods of allocating participants to a treatment were not all strictly randomized (e.g., hospital record number, order of admission, and day of the week). Also, the method of data analysis was not specified in three studies. Different outcome parameters and methods of reporting the results were used. Consequently, interesting parameters could not be analysed, for example, the 30-day mortality. In addition, the studies meeting the inclusion criteria were individual trials with a small sample size without an adequate power calculation.

The total number of available randomized trials is still small, however they jointly involve almost 1,000 patients. Although definitive conclusions cannot be drawn from these results, there seems to be a more prominent and beneficial role for total hip arthroplasty over hemiarthroplasty in the growing group of selected patients with femoral neck fractures.

### Implications for future research

Although there is a growing awareness of the possibility of better results for selected patients treated with THA for displaced femoral neck fractures, a randomized trial is needed to definitively answer this long-lasting controversy in trauma surgery. One such unique international collaborative initiative (IHFRC; www.ihfrc.ca) is currently actively enrolling patients in a multinational trial comparing revision surgery, functional outcome and quality of life after THA versus HA in elderly patients who sustained a displaced femoral neck fracture [31]. This study would allow further assessment of the clinical relevance of the relatively small differences in pain and functional outcome found in the present study. This trial is important because it has the potential to substantially change surgical practice for the management of femoral neck fractures [32].

## Conclusion

This review, including the most recent evidence, shows that total hip arthroplasty may be advantageous over hemiarthroplasty in a selected group of patients suffering displaced femoral neck fractures. Ultimately, only large, well-designed and well-conducted studies will result in improvements in the outcomes of treatment and resolve the longstanding controversy of whether total hip arthroplasty or hemiarthroplasty is the preferred treatment modality for this common fracture.

## Appendix 1: Last searches carried out on 22 February 2011

### PubMed: *N* = 211

(hip fractures[mesh] OR hip fracture*[tw] OR femoral neck fractures[mesh] OR femoral neck fracture*[tw] OR femur neck fracture*[tw] OR femoral collum fracture*[tw] OR femur collum fracture*[tw] OR intracapsular hip fracture*[tw] OR subcapital hip fracture*[tw] OR intracapsular collum fracture*[tw] OR subcapital collum fracture*[tw] OR intracapsular neck fracture*[tw] OR subcapital neck fracture*[tw]) AND (arthroplasty[mesh] OR arthroplast*[tw] OR hemiarthroplast*[tw] OR hip replace*[tw] OR hip prosthe*[tw]) AND random*[tw] NOT (animals[mesh] NOT humans[mesh])

### EMbase: *N* = 121

('femur neck fracture'/syn OR (('femoral neck' OR 'femur neck' OR 'femoral collum' OR 'femur collum' OR 'intracapsular hip' OR 'subcapital hip' OR 'intracapsular collum' OR 'subcapital collum' OR 'intracapsular neck' OR 'subcapital neck') NEAR/3 fracture*):ti,ab,de) AND ('hip arthroplasty'/syn OR hemiarthroplast*:ti,ab,de OR (hip NEAR/3 (replace* OR prosthe*)):ti,ab,de) AND random*:ti,ab,de NOT (animal/de NOT human/de)

### WoS: *N* = 774

(hip fracture* OR femoral neck fracture* OR femur neck fracture* OR femoral collum fracture* OR femur collum fracture* OR intracapsular hip fracture* OR subcapital hip fracture* OR intracapsular collum fracture* OR subcapital collum fracture* OR intracapsular neck fracture* OR subcapital neck fracture*) AND (arthroplast* OR hemiarthroplast* OR hip replace* OR hip prosthe*) AND random* NOT (animal* NOT human*)

## Appendix 2

**Table:**

Minor complications included all reported cases of:
Anemia
Ileus
Superficial wound infect
Urinary tract infection
Deep venous thrombosis
Blood transfusion
Atrial fibrilation
Pneumonia
Decubitus
Heart failure
Postoperative confusion
Other infection
Major complications included all reported cases of:
Myocardial infarction
Deep infection
Stroke
Pulmonary embolism
Sepsis
Hematemesis/ GI bleeding
Re-operation (not revision)
